# Global Workforce Development in Father Engagement Competencies for Family-Based Interventions Using an Online Training Program: A Mixed-Method Feasibility Study

**DOI:** 10.1007/s10578-021-01282-8

**Published:** 2021-11-20

**Authors:** Vilas Sawrikar, Alexandra L. Plant, Brendan Andrade, Matt Woolgar, Stephen Scott, Eli Gardner, Celia Dean, Lucy A. Tully, David J. Hawes, Mark R. Dadds

**Affiliations:** 1 Department of Clinical Psychology, School of Health in Social Science, University of Edinburgh, Edinburgh, UK; 2 Centre for Addiction and Mental Health, Toronto, ON Canada; 3 Department of Psychiatry, University of Toronto, Toronto, ON Canada; 4 Department of Child and Adolescent Psychiatry, Institute of Psychiatry, King’s College London, London, UK; 5 Kidsmatter, London, UK; 6 School of Psychology, Faculty of Science, University of Sydney, Sydney, NSW Australia

**Keywords:** Father engagement, Practitioner training, Practitioners, Confidence, Competence

## Abstract

Global access to practitioner training in the clinical engagement of fathers in family-based interventions is limited. The current study evaluated the feasibility of training practitioners in Canada and UK using online training developed in Australia by examining improvements in practitioner confidence and competence in father engagement, training satisfaction, qualitative feedback, and benchmarking results to those from an Australian sample. Practitioners were recruited to participate in a 2-h online training program through health services and charity organisations. The online program required practitioners to watch a video and complete self-reflection exercises in a digital workbook. Pre- and post-training measures were collected immediately before and after the online training program. The results indicated significantly large improvements in self-reported confidence and competence in engaging fathers following training, with levels of improvement similar to those found in Australia. Training satisfaction was high and qualitative feedback suggested providing local resources and increasing representation of social diversity could improve training relevance in local contexts. The findings suggest online training in father engagement can contribute to global workforce development in improving practitioners’ skills in engaging fathers in family-based interventions.

**Supplementary Information:**

The online version contains supplementary material available at 10.1007/s10578-021-01282-8.

## Introduction

Research shows that fathers play an important role in their child’s development [1]. However, a significant gap exists between recognition of the importance of fathers in child development and representation of fathers in attendance and participation in child mental health treatments [2–4]. This is especially problematic in family-based interventions for child behaviour problems where involving fathers in treatment could enhance child outcomes [5, 6]. Practitioner’s competencies to engage fathers have become a target of workforce development to improve father engagement in parenting programs [7]. Recent findings from an Australian study provided preliminarily support for online training as an effective method of increasing practitioners’ confidence and competence in relation to engaging fathers, and disseminating training on a large scale [8]. In line with this, the current study evaluated the feasibility of global workforce development in father engagement by examining training effectiveness and satisfaction when online training is provided to practitioners working in international contexts (Canada and UK).

A large and convincing evidence base exists for the effectiveness of family-based interventions in supporting children with a range of child problems such as disruptive behaviour and anxiety problems [5, 9–12]. However, research shows that parents’ treatment engagement behaviours like attendance and quality of participation are important for effective treatment [13–16]. Furthermore, findings from meta-analyses show improvements from a family-based intervention for child behaviour problems are greater for the child when both mothers and fathers participate in treatment compared to when mothers participate in treatment alone [6, 17]. And individual studies have also demonstrated the positive impact of father involvement for parent and child outcomes in family-based interventions targeting infant-parent relationships, eating disorders, and challenging behaviours in context of intellectual disability across different countries [18–22].

Despite the importance of father participation in family-based interventions, international research examining parental engagement suggests fathers are less likely to participate and benefit from family-based interventions [23–26]. Even when both parents are invited to participate, fathers’ rate of attendance and quality of participation tend to decline across the intervention [18, 27]. Preliminary results from qualitative research suggest barriers to treatment engagement may be experienced differently by fathers. For instance, research suggests that fathers’ attitudes around gender roles and help‐seeking, fathers’ low knowledge of family-based interventions, programs not meeting fathers’ individual needs (e.g., managing personal and parenting stress), and the perception that interventions are directed towards mothers, may be key barriers to engaging fathers in family-based interventions [28, 29]. These findings suggest a need to develop father-inclusive approaches to the design and delivery of family-based interventions to improve the effectiveness of treatment [2, 30].

Practitioners notably represent an important target for enhancing father engagement with specific attention to training clinical–interpersonal skills in engaging parents [2, 8, 31, 32]. Indeed, reviews of research findings from different countries suggest there are significant limitations in practitioner’s knowledge and confidence to engage fathers in family-based interventions [2, 33]. Surveys of mental health practitioners indicate that, while many endorse father participation in treatment as important, most of them report low levels of competence and use of strategies to engage fathers [7]. Practitioners also report limited knowledge of methods to engage fathers in family-based interventions [34]. Further adding to arguments for the need to target practitioners is international evidence that few receive specific training in engaging fathers within tertiary level courses in health, welfare, and education [34, 35]. Collectively, these findings suggest a need for developing and widespread dissemination of practitioner training programs in father engagement. Previous studies evaluating face-face training in father engagement competencies have shown positive results in potentially improving practitioners’ self-reported use of father engagement skills [36–38].

While results related to face-face training are positive, online training represents a low-cost method to overcome barriers to training practitioners on a large scale. For instance, research suggests that a major barrier to training practitioners is the cost and resource burden to health professionals and services [2, 39]. Face-face training places significant demands on practitioners’ time which in turn may reduce uptake of training [8]. Practitioners’ participation in training can also disrupt service delivery due to reduced staff availability because of time allocated to training leading to lack of organisational support [40]. Direct costs in delivering training would be avoided via online training formats since training would not require employment or availability of trainers. Further, practitioners can access training at their convenience, thus limiting disruption to service delivery. Research supports online training methods to achieve similar effects as face-face delivery in training health professionals [41]. Further, the increased accessibility of training via the internet means that online training has the potential for widespread dissemination and reach into communities [8]. This includes increasing reach to practitioners in overseas health settings as there are no geographic boundaries associated with online training delivery.

Previous research evaluating online practitioner training in father engagement competencies showed promise in improving practitioners’ use of father engagement skills in local contexts. Drawing from social cognitive theory, Burn et al. [8] evaluated a training program delivered in either face-face or online formats. This training targeted practitioners’ confidence and competence in using strategies to engage fathers, as well as organisational practices to engage fathers, and was tested in a diverse sample of mental health professionals in Australia. Both training formats were associated with improvements in practitioner competencies, organizational practices, and self-reported rates of father engagement over time. Practitioners reported high levels of satisfaction with both program formats. While some deterioration in self-reported competencies was noted from post-training to three-month follow-up for practitioners in the online format, the follow-up scores were still significantly improved compared with pre-training. This research demonstrates the potential for online training programs targeting practitioners’ self-efficacy in engaging fathers to improve rates of father attendance in family-based interventions. The preliminary evidence also shows promise in overcoming putative barriers for practitioners to access training. No research exists evaluating training programs disseminated internationally to address low father engagement in family-based interventions on a global scale.

Following Kirkpatrick and Kirkpatrick’s [42] training evaluation framework, the current study examined the feasibility of disseminating the Australian-based online training program [8] internationally by examining training effectiveness and satisfaction, as well as qualitative feedback among child and family practitioners in the United Kingdom (UK) and Canada. Based on Burn et al. [8], it was hypothesised that the training would be effective, and practitioners’ confidence and competence would improve following participation in the online training program (Hypothesis 1). Benchmarking analysis was used to compare previous training outcomes from Australia [8] to those in the UK to evaluate equivalency in training effectiveness. It was expected that training effectiveness would be equivalent between practitioners in the UK and Australia (Hypothesis 2). We also examined practitioners’ self-reported satisfaction with the training program. Finally, thematic analysis was used to analyse practitioners’ feedback about the training program. The aim of the qualitative analysis was to evaluate practitioners’ reports of the quality of, and satisfaction with, the training program, as well as identify future directions for ensuring the program is appropriate for different countries.

## Method

### Study Design and Procedures

The current study consisted of a single group design evaluating pre- to post-training changes in practitioners’ competency and skills in engaging fathers. Health service organisations that delivered child and family services in the UK and Canada were approached to assist with recruiting practitioners prior to the start of the study. UK health services assisting this study included the South London and Maudsley National Health Service located in South East London region, while the primary health service organisation participating in this study from Canada was the Center for Addition and Mental Health located in Toronto, Ontario. The Children and Young Peoples Improving Access to Psychological Therapies (CYP IAPT) program supporting practitioner training at the UK site also agreed to assist with recruitment for the current study. The CYP IAPT is a National Health Service (NHS) program designed to improve the quality and access of services for children and young people [43]. Finally, practitioners could access the training program directly via a dedicated training website. Recruitment into the study in the UK and Canada was open from February 2019 to July 2020. The Australian sample of practitioners included for the benchmarking analysis was the same analysed in Burn et al., ([8]; refer to study for details of Australian participants).

The Human Research Ethics Committee (HREC) at the University of Sydney, Australia, provided ethical approval for this study, while local ethics committee approvals from Kings College London and Center for Addiction and Mental Health were obtained for recruiting participants within organisations in the UK and Canada. Prior to online training, practitioners were able to read an information sheet where they were notified that participation was anonymous and voluntary, as well as options to withdraw at any time without consequence. Practitioners interested in taking part in the study provided consent by clicking on the statement “I agree, start the training program” using the Qualtrics ™ online survey platform.

Participants were directed to complete pre-training measures after providing informed consent and then directed to the online training package where they viewed an online video and completed a digital workbook (see content of online training program section below). Participants completed the post-training questionnaires immediately after finishing the online training package. Participants did not have to respond to items assessing self-reported confidence and competence in engaging fathers in pre- and post-training assessments if they did not work directly with families. The anonymous dataset was downloaded from Qualtrics to a secure, password-protected network drive for analysis at the end of the study.

### Content of Online Training Program

Details of the online ‘Engaging Fathers in Parenting Programs’ training program can be found in Burn et al. [8]. In summary, the training program was based on social cognitive theory designed to increase practitioners’ self-efficacy to engage fathers and ability to self-evaluate practitioners’ own learning regarding father engagement skills, in order to modify implementation of clinical–interpersonal skills in engaging fathers. The online program was a 50-min training video consisting of five topics; (1) research background, (2) barriers to engaging fathers, (3), positive engagement strategies, (4), managing parental conflict, and (5) planning for future father-inclusive practices (see Fig. 1 in supplementary information). Video included didactic presentations, as well as vignettes of practitioners and families to demonstrate key skills. Participants were asked to download a digital workbook to their personal devices prior to starting the training where they were able to write notes while watching the video. The workbook was developed in portable document format (PDF) with text box fields to allow participants to record and save electronic notes. Workbook tasks were designed to increase self-awareness of father engagement skills and reinforce skills acquisition through reflective learning [8]. Responses to tasks were not self-assessed during online training. The training program was designed to take 2 h to complete.

**Fig. 1:**
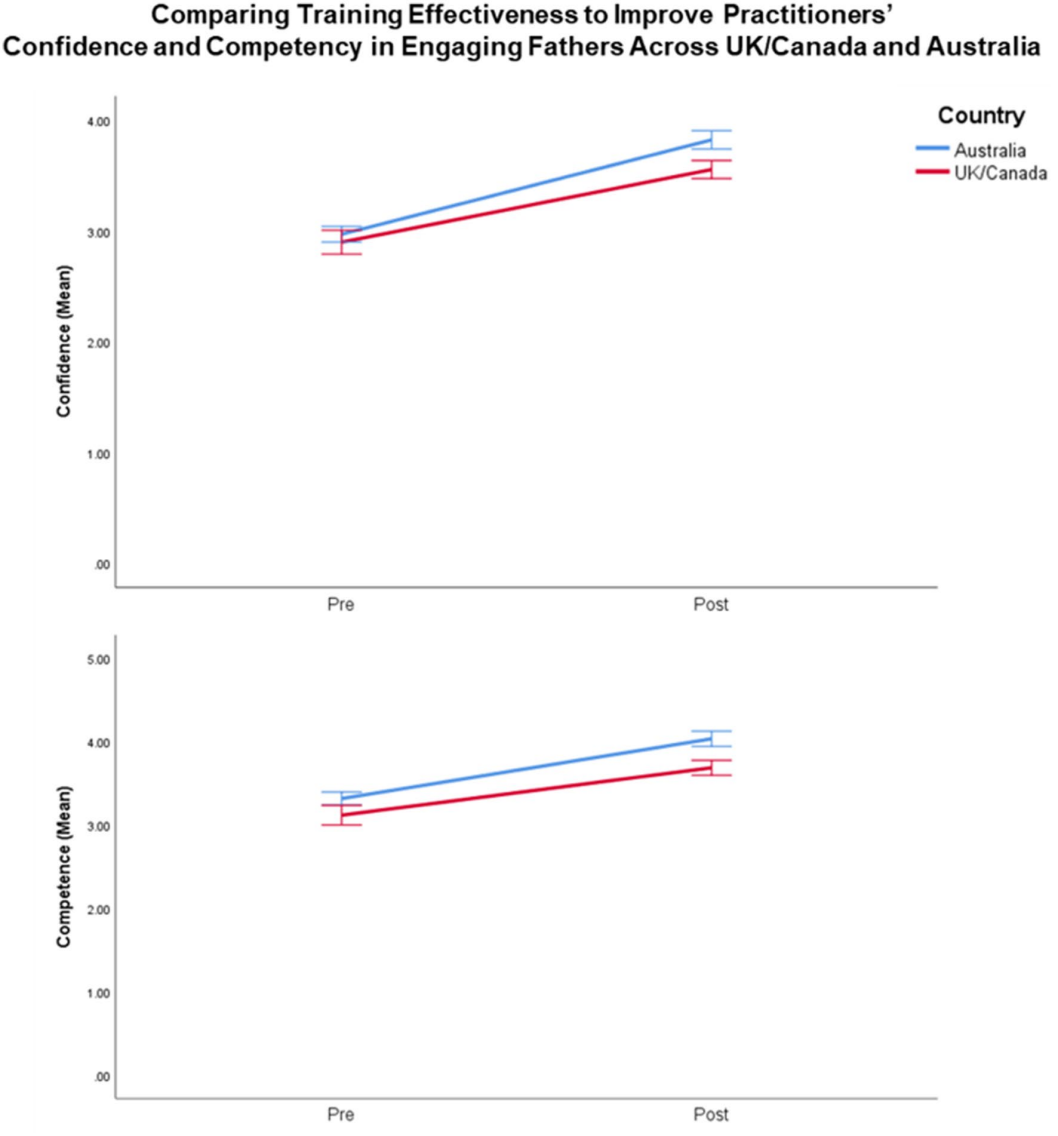
Benchmarking analysis comparing training effectiveness across the UK and Australia

### Participants

Eligibility criteria for practitioners to be included in the study were: practitioners working for a child and family organisation or delivering family-based interventions for child behaviour problems, aged 18 or over, and residing in the UK or Canada. Non-health professionals (e.g., teachers, support staff, health service managers) were also eligible to participate contingent on working within a child and family organisation. Practitioners were invited to take part in the study via an email flyer distributed using organisation email lists. The email directed practitioners to the training website for further information about the study where they could read the information sheet and complete consent, pre-training measures, online training, and post-training measures. The UK sample had 229 practitioners provide consent and complete pre-training questionnaires, with 223 participants proceeding to completing post-training questionnaires (97.4%). The Canadian sample had 45 practitioners provide consent and complete pre-training questionnaires, with 17 participants proceeding to complete post-training questionnaires (37.8%). Survey response rates are unknown as details of number of practitioners recruited via email flyers versus directly through the website was not recorded.

### Measures

#### Sociodemographic Information

Sociodemographic questions related to gender, profession, organisation type, years of experience working with families and, experience of training in father engagement. Responses to the item assessing profession were coded to identify groups of health and care professionals across countries for the benchmarking analysis (see Table 1 in supplementary information for coding scheme). Coding was completed by two independent raters with high agreement (κ = 0.91).

**Table ﻿1:** Characteristics of participants included versus excluded in the analysis examining training effectiveness

Participant characteristic	United Kingdom		Canada	
	Included	Excluded	Included	Excluded
Gender (*N*)				
Female	167 (91.3%)	38 (90.5%)	17 (100%)	24 (85.7%)
Male	16 (8.7%)	4 (9.5%)	0 (0%)	4 (14.3%)
Years of professional experience (*M*, SD)	8.59 (8.83)	7.14 (8.40)	8.12 (9.13)	12.75 (9.48)
Profession (*N*)				
Practitioner psychologist	117 (64.3%)	26 (61.9%)	9 (52.9%)	7 (25.0%)
Social Worker	8 (4.4%)	3 (7.1%)	3 (17.6%)	15 (53.6%)
Counsellor/caseworker/family support worker	25 (13.7%)	7 (16.7%)	2 (11.8%)	1 (3.6%)
Nurse	7 (3.8%)	1 (2.4%)	1 (5.9%)	0 (0.0%)
Medical or allied health practitioner	3 (1.6%)	0 (0.0%)	0 (0%)	1 (3.6%)
Manager/administration	5 (2.7%)	0 (0.0%)	0 (0%)	1 (3.6%)
Other	17 (9.3%)	2 (4.8%)	2 (11.8%)	2 (7.1%)
Organisation type (*N*)				
Child and adolescent mental health service	113 (61.7%)	29 (69.0%)	16 (94.1%)	17 (60.7%)
Other government service	23 (12.6%)	4 (9.5%)	0 (0.0%)	6 (21.4%)
Non-government organisation	13 (7.1%)	4 (9.5%)	0 (0%)	0 (0%)
University-based clinic	2 (1.1%)	0 (0.0%)	0 (0%)	0 (0%)
Private practice	2 (1.1%)	0 (0.0%)	0 (0%)	0 (0%)
Other	30 (16.4%)	5 (11.9%)	1 (5.9%)	5 (17.9%)
Directly working with families (*N*)				
Yes	178 (97.3%)	21 (50.0%)	17 (100%)	28 (100%)
No	5 (2.7%)	21 (50.0%)	0 (0%)	0 (0.0%)
Previous training in father engagement (*N*)				
Yes	17 (9.3%)	7 (16.7%)	1 (5.9%)	6 (24.4%)
No	166 (90.7%)	35 (83.3)	16 (94.1%)	22 (78.6%)

Practitioner psychologist included Psychologist, Psychological Wellbeing Practitioner, and Education Mental Health Practitioner (based on UK Health and Care [HCPC] Professional groups); Medical and allied health practitioner included Psychiatrist, Paediatrician, General Practitioner, Occupational Therapist, Speech Pathologist, and Physiotherapist

#### Practitioners’ Competence and Confidence in Engaging Fathers

The Father Engagement Questionnaire (FEQ; 44) was used to assess practitioners’ self-reported competence and confidence in engaging fathers. The confidence scale consisted of nine items assessing practitioner’s confidence in engaging fathers in treatment (e.g., ‘How confident do you feel in engaging fathers who are reluctant to attend’). The competence scale consisted of five items assessing how competent practitioners felt in implementing strategies to engage fathers (e.g., ‘To what extent do you feel competent to listen reflectively and creating shared understanding about both parents’ perspectives, even when they differ’). Both scales were rated on a 5-point Likert scale from ‘not at all confident/competent’ [1] to ‘extremely confident/competent’ [5]. Total scores were based on average scores across items. Participants had the option to indicate ‘do not work directly with families’ [8]. The internal consistency for the confidence and competency scales were high in the current study (UK: α = 0.92; Canada: 0.92).

#### Satisfaction with the Training Program

Training satisfaction was assessed using the Questionnaire for Professional Training Evaluation (Q4TE) in relation to scales of Reaction and Learning [45]. The Reaction scale consisted of two, two-item subscales of satisfaction and utility. The Learning scale consisted of one, two-item subscale of knowledge. A further two questions were added to the Q4TE to assess participants’ overall satisfaction of the training and their intent to utilise the training, with the latter item included as a proximal predictor of application of skills into practice [46]. Participants rated their level of agreement to each item statement on a 11-point Likert-scale ranging from 0 percent (‘completely disagree’; coded as 0) to 100 percent (‘completely agree’; coded as 10) with single steps representing 10 percent increase.

### Analytics plan

All quantitative analyses were conducted in IBM SPSS version 25. Characteristics of participants were summarised using descriptive statistics (mean, standard deviation) in preliminary analysis. Comparisons between participants included versus excluded (due to missing information or participant opting not to provide response to FEQ items) from analyses examining training effectiveness were evaluated to determine whether missing information may influence estimates of training effects. Comparisons were examined using Independent T-tests for continuous variables and Chi square analysis for categorical variables.

Training effectiveness was evaluated by significance of pre- to post-training changes in self-reported competencies and confidence in engaging fathers using repeated measures multivariate analysis of variance (MANOVA). Training effects were analysed separately for the UK and Canada. Self-reported measures of practitioner competence and confidence were entered into the model as dependent variables, while Time (pre- and post-training) was entered as the main repeated measures factor. Significant results were followed by an examination of univariate main effects for time. Significant effects were evaluated at α = 0.05 and effect sizes measured by partial eta-squared (η^2^_*p*_) were considered either small, 0.01, moderate, 0.09, or large, 0.25 [47].

In the UK and Canada sample, satisfaction with training was assessed by examining descriptive statistics (mean, standard deviation, median, interquartile range) of responses to the Q4TE summarised at both individual item-level (eight questions), Q4TE subscales (Satisfaction, Utility, Knowledge), Q4TE scales (Reaction, Learning), as well as items assessing overall satisfaction and intent to utilise the training. Qualitative analysis evaluating training satisfaction and experience among UK and Canadian practitioners (combined) was conducted using inductive thematic analysis. Qualitative reports of training experience were gathered from written responses to three open ended questions inviting feedback about the quality of and satisfaction with the training program, including future recommendations for developing the training program for their local context (‘Q1. Were there any aspects of the training workshop that were helpful’, ‘Q2. Were there any aspects of the training workshop that weren’t helpful’, ‘Q3. How would you recommend to adapt the program for the [UK] or [Canada] context’). Qualitative responses were analysed using Braun & Clark’s [48] six-step inductive procedure for thematic analysis. Responses were read and coded, checked and grouped together to identify themes and sub-themes. The coding system was developed by the second author (AP) and verified in discussion with the first author (VS). Coding of responses was completed by one rater (AP).

Finally, benchmarking analysis evaluated outcomes between health and care professionals recruited in the UK and Canada versus Australia for comparability. The UK and Canada groups were combined into a single ‘overseas’ group given the small sample size at the Canada site. The benchmarking analysis began with comparing demographic information (gender, years of experience, previous training) and pre-training scores between the Australian and overseas samples. MANOVA tested equivalency in training effectiveness by level of significance of the interaction Country × Time. Significant interaction effects indicated non-equivalence in training effectiveness. Significant effects were evaluated at α = 0.05.

## Results

### Preliminary Analyses

Table 1 summarises the characteristics of participants included versus excluded in the analysis examining training effectiveness. For the UK sample, most participants included in the analysis were female (91.3%) working directly with families (97.3%) in a child and adolescent mental health service (61.7%). Further, most participants were practitioner psychologists (64.3%), followed by counsellor/caseworker/family support worker (13.7%), and social workers (4.4%). Most participants reported never receiving training in working with fathers (90.7%). Years of professional experience varied from 0 – 40 years, with average years of experience of 8.59 (SD = 8.83) years and modal years of experience of less than 1 year (10.4%). For the Canada sample, participants included in the analysis were female (100%) working directly with families to deliver treatments (100%) in a child and adolescent mental health service (94.1%). Most had never received training in working with fathers (94.1%). Participants were generally practitioner psychologists (52.9%). Years of professional experience varied from 2 to 31 years, with average years of experience of 8.12 (SD = 9.13) years and modal years of experience of 4 years (23.5%).

UK participants included in the analysis examining training effectiveness were similar to those excluded in relation to gender, χ^2^(1) = 0.03, *p* = 0.87, profession, χ^2^(9) = 3.69, *p* = 0.82, previous training in engaging fathers, χ^2^(1) = 1.95, *p* = 0.16, and years of professional experience, *F*(1,221) = 0.93, *p* = 0.34, as well as self-reported confidence, *F*(1,202) = 1.58, *p* = 0.21, and competence, *F*(1,202) = 1.60, *p* = 0.21, in engaging fathers. Likewise, Canadian participants included in the analysis examining training effectiveness were similar to those excluded in relation to gender, χ^2^(1) = 2.67, *p* = 0.10, profession, χ^2^(7) = 10.52, *p* = 0.16, previous training in engaging fathers, χ^2^(1) = 1.95, *p* = 0.16, and years of professional experience, *F*(1,43) = 2.60, *p* = 0.12, as well as self-reported confidence, *F*(1,42) = 0.01, *p* = 0.92, and competence in engaging fathers, *F*(1,42) = 0.44, *p* = 0.51.

### Evaluating Training Effectiveness in UK and Canada

Practitioners’ self-reported competency and confidence at the pre- and post-training assessments for practitioners recruited from the UK and Canada are summarised in Table 2. In the sample of UK practitioners, results from the repeated measures MANOVA indicated significant multivariate main effects for Time, *F* (2, 177) = 111.97, *p* < 0.001, Hotelling’s Trace = 2.04, η^2^_*p*_ = 0.56. Follow-up univariate tests indicated significant increases in both confidence, *F* (1, 178) = 217.97, *p* < 0.001, η^2^_*p*_ = 0.55, and competence, *F* (1, 178) = 125.69, p < 0.001, η^2^_*p*_ = 0.41. Training effect sizes were in the large range. In the sample of Canadian practitioners, results from the repeated measures MANOVA indicated significant multivariate main effects for Time, *F* (2, 15) = 14.14, *p* < 0.001, Hotelling’s Trace = 1.89, η^2^_*p*_ = 0.65. Follow-up univariate tests indicated significant increases in both confidence, *F* (1, 16) = 18.28, *p* < 0.001, η^2^_*p*_ = 0.53, and competence, *F* (1, 16) = 30.12, p < 0.001, η^2^_*p*_ = 0.65. The results indicate that the online training program was associated with improvements in practitioners’ confidence and competence in father engagement for both samples of UK and Canadian practitioners.

**Table 2:** Pre- and post-training ratings of practitioners’ Confidence and Competence in engaging fathers

	Pre-training	Post-training
	Mean (SD)	Mean (SD)
*United Kingdom*		
Confidence	2.86 (.65)	3.60 (.54)
Competence	3.08 (.70)	3.73 (.58)
*Canada*		
Confidence	3.15 (.45)	3.63 (.52)
Competence	3.15 (.60)	3.78 (.56)

*SD* standard deviation

### Training Satisfaction

Table 3 summarises the descriptive statistics for items and scales assessing practitioners’ satisfaction with online training. For participants in the UK, average item-level ratings ranged from 70.8% (‘I enjoyed the training very much) to 80.3% (‘I intend to use the knowledge I gained in the training in my everyday work’). Median item-level ratings were all above 75%, except for items assessing Q4TE Satisfaction scale which was 72.5%. Average scale-level ratings ranged from 72.8% (Satisfaction scale) to 79.1% (Utility scale). Median scale-level ratings for Reaction and Knowledge/Learning scales, as well as the subscale of Utility, were all above 75%, while the median scale-level ratings for Satisfaction was 72.5%. UK participants reported high levels of intent to use the knowledge in their everyday work (mean: 80.3%; median: 80.0%) and overall satisfaction with the program (mean: 80.0%; median: 80.0%). For participants in Canada, average item-level ratings ranged from 78.8% (‘I learned a lot of new things in the training’) to 92.4% (‘Participation in this kind of training is very useful for my job’). Median item-level ratings were all above 75%. Average scale-level ratings ranged from 80.0% (‘Knowledge Scale’) to 90.0% (‘’ Utility Scale’). Median scale-level ratings for Reaction and Knowledge/Learning, as well as subscales of Satisfaction and Utility, were all above 75%. Canadian participants reported high levels of intent to use the knowledge in their everyday work (mean: 88.8%; median: 90.0%) and overall satisfaction with the program (mean: 85.3%; median: 90.0%).

**Table 3:** Descriptive statistics for scales assessing practitioners’ satisfaction with the online training program

Country	Subscale	Item	UK (*n* = 204)			Canada (*n* = 17)		
Scale			Mean (SD)	Median	Interquartile range	Mean (SD)	Median	Interquartile range
Reaction	Satisfaction	I will keep the training in good memory	74.4 (18.2)	70.0	60.0 – 80.0	84.1 (15.0)	90.0	75.0 – 100
		I enjoyed the training very much	70.8 (20.2)	70.0	70.0 – 90.0	80.0 (14.1)	80.0	70.0 – 90.0
		Total Satisfaction Scale	72.8 (18.5)	72.5	65.0 – 85.0	82.1 (14.0)	90.0	72.5 – 90.0
	Utility	The training is very beneficial to my work	78.5 (19.6)	80.0	70.0 – 100	87.6 (9.0)	90.0	80.0 – 95.0
		Participation in this kind of training is very useful for my job	79.5 (19.5)	80.0	70.0 – 100	92.4 (9.0)	90.0	90.0 – 100
		Total Utility Scale	79.1 (18.7)	80.0	70.0 – 95.0	90.0 (8.3)	90.0	85.0 – 97.5
		Total Reaction Scale (Satisfaction, Utility)	76.0 (17.9)	77.5	67.5 – 90.0	86.0 (10.6)	90.0	76.3 – 95.0
Learning	Knowledge	After the training, I know substantially more about the training contents than before	77.7 (19.5)	80.0	70.0 – 90.0	81.1 (16.9)	80.0	65.0 – 100
		I learned a lot of new things in the training	74.2 (20.5)	80.0	60.0 – 90.0	78.8 (16.5)	80.0	65.0 – 95.0
		Total Knowledge Scale	76.1 (19.3)	80.0	65.0 – 85.0	80.0 (16.2)	80.0	65.0 – 95.0
Additional items		Overall, I am satisfied with the training	80.0 (19.9)	80.0	70.0 – 100	85.3 (12.8)	90.0	75.0 – 100
		I intend to use the knowledge I gained in the training in my everyday work	80.3 (19.8)	80.0	70.0 – 90.0	88.8 (12.2)	90.0	80.0 – 100

### Qualitative Thematic Analysis Examining Practitioner Feedback About Training Program

Q1 had 178 responses, Q2 had 126 responses, and Q3 had 122 responses. Three main themes were identified: (i) delivery method and format, (ii) training content, and (iii) future training development (see Table 2 in supplementary information). Most themes mapped onto positive and negative feedback given questions were framed to invite comments about the helpful and unhelpful aspects of the training program.

#### Delivery Method and Format

A subtheme of positive feedback was *combination of resources* reflecting that participants enjoyed the combination of videos and workbook throughout the training, which participants found helpful and easy to access. Another subtheme was *helpful role play videos* whereby participants reported that it was helpful to see engagement strategies in the videos. It was suggested that the use of videos encouraged practitioners to reflect on their own practice which increased their confidence in using the recommended engagement strategies. A subtheme of negative feedback was that the *format created challenges to engage* whereby participants felt overwhelmed by the amount of information presented, the length of the online training, and difficulties in concentrating. A theme of *preferring face-face training* was also identified reflecting potential different learning styles and preferences.

#### Session Content

A subtheme of positive feedback was *usefulness of engagement strategies*, with specific reference to strategies for establishing a parenting team in practice and conflict management. Another subtheme of positive feedback was *implications for practice*, whereby participants reflected how the training encouraged reflective practice that would have a positive benefit for their future work with fathers. By contrast, a subtheme of negative feedback was *content development*, whereby participants stated the content should be expanded further, suggesting they felt they wanted more from the program.

#### Future Training Development

Practitioners proposed directions for future development under the theme of *methods of adapting training,* whereby practitioners suggested providing references to local information and resources. *Diversity* was another identified subtheme whereby participants reported that a more diverse group of fathers should be discussed/shown in the videos, including different cultures, religious beliefs, race and socioeconomic status, as well as diversity in the couples observed, such as same sex couples.

### Benchmarking Analysis

There were no significant differences between sample groups (*N*_Australia_ = 171; *N*_overseas_ = 178) on measures of years of experience (*t* (315) = -0.62, *p* = 0.54), gender (*x*^*2*^ (1) = 2.76, *p* = 0.10), and self-reported confidence in engaging fathers (*t* (347) = 1.68, *p* = 0.10). However, significant differences were found for previous training of father engagement (*x*^*2*^ (1) = 10.30, *p* = 0.00) and self-reported competence in engaging fathers (*t* (347) = 3.78, *p* = 0.00), whereby the Australian sample had more participants receiving previous training in father engagement (Australia: 20.5%; Overseas: 8.4%) and higher self-reported competence in engaging fathers at baseline compared to overseas participants (*M*_Australia_ = 3.36, *SD* = 0.64; *M*_overseas_ = 3.09, *SD* = 0.68). The final model included previous training in father engagement as a covariate. The repeated measures MANOVA did not yield significant interaction effects, *F* (2, 346) = 2.43, Hotelling’s Trace = 0.01, *p* = 0.08 (Fig. 1), indicating that the changes in self-reported competency and confidence in engaging fathers were similar for overseas and Australian practitioners.

## Discussion

The current study examined whether it was feasible to educate health practitioners in father engagement competencies on a global scale using online training. Samples of health practitioners from the UK and Canada were used as case examples. Feasibility was assessed by training effectiveness, satisfaction, and qualitative feedback regarding practitioners’ experience of the training program. Providing support for hypothesis 1, the results indicated significantly large improvements in self-reported confidence and competence in engaging fathers following participation in the online training program among practitioners from the UK and Canada. Providing support for hypothesis 2, rates of improvements in self-reported confidence and competence among UK and Canadian practitioners were similar to that observed in a previous Australian study [8]. Self-reported ratings of training satisfaction were high overall. Qualitative feedback also indicated high satisfaction with the training program, while providing directions of future development in relation to training content. Overall, the findings indicate that it is possible to disseminate an online training program in father engagement into overseas health settings.

Significantly large improvements in practitioners’ self-reported confidence and competency in engaging fathers suggests that, the online training program is effective in increasing practitioners’ self-efficacy in the use of skills to engage fathers in family-based interventions. The results extend and replicate research findings showing that training in father engagement competencies improves domains of self-efficacy considered important to application of engagement skills when training is delivered in online formats, at least when self-efficacy was measured immediately after participation in online training [8, 36–38]. Importantly, the current study showed training effectiveness in samples of practitioners working in different countries from which the original program was developed with similar levels of training effectiveness. The findings also provided further support that this training program was effective for a range of professionals working in different countries and settings. Overall, the results suggest that the online training program can be effective in improving father engagement competencies when disseminated into different countries.

Practitioners self-reported high levels of satisfaction with the online training program, in relation to their satisfaction, utility, and knowledge, converges with previous evaluation studies of father engagement competency training [8, 36, 38]. Practitioners in the current study also reported high levels of intent to use the knowledge in their everyday work suggesting translatability of training knowledge into practice, although the current study did not measure whether training participation led to measurable change in practitioners’ use of engagement skills. Qualitative analysis provided further support for training satisfaction among health practitioners in the UK and Canada. The findings suggested that the online training program was well-received by health practitioners in the UK and Canada in relation to delivery and format of training, as well as training content and implications for practice. However, minimal adaption might be required to make the program relevant to local contexts. It is noted that, while findings for satisfaction and qualitative themes were consistent, the Canadian site had higher rates of incomplete post-training measures, thus finding are somewhat tentative.

Taken together, the results preliminarily support the feasibility of using online training as part of global workforce development improving father engagement competencies. However, practitioners’ feedback may be useful in refining approaches to dissemination. Practitioners’ suggestions to localise resources and increase social diversity in training content to represent local populations are well taken to improve training relevance to local contexts. Previous studies have shown the value of adapting content to improve outcomes for healthcare professionals [49]. However, these suggestions should be considered in context of practitioners’ reports that the standard online program improved self-efficacy in engaging fathers, was suitable for their context, and high satisfaction with the program. Furthermore, consideration should be given to costs in adapting content as the online format was intended to provide low-cost methods of training [8]. Rather than adapting content therefore, it might be helpful to consider how best to implement online training. Blended learning models whereby online training is delivered alongside other learning modules is a well-established method of integrating online learning for healthcare workforce development [50]. To this end, we propose that access to follow-up support such as reflective practice workshops or cultural competence training be provided to consider cultural variation [51].

Discrepancies in completion rates of post-training measures across the UK and Canadian samples may also be important in disseminating the online program. It important to first note that low completion of post-training measures may not apply to rates of completing the actual training. Nevertheless, investigations were conducted during the study to determine whether noncompletion in the Canadian sample was associated with differences in instructions or website design, but neither were found to explain noncompletion. We hypothesise that the difference was related to structural differences in how the two cohorts were engaged. While recruitment at both sites involved the use of emails lists distributed within child and family services, recruitment in the UK included email lists of practitioners in the local CYP IAPT program. The CYP IAPT program delivers post-graduate training to practitioners for improving service quality. The higher completion rates in the UK may therefore relate to the utilisation of a specialised network for practitioner training. A key implication for further analysis is whether effective dissemination could be maximised with the support of existing training networks in local settings.

Several limitations of the current study should also be noted as areas of further research in disseminating the online ‘Engaging Fathers in Parenting Program’ internationally. First, the current feasibility analysis only recruited a small number of child and family organisations from the UK and Canada. Non-completion of post-training questionnaires in Canada was also high, putatively leading to low power in examining outcomes; however, findings were notably consistent. Nonetheless, future research should particularly examine feasibility of disseminating the online training program in different organisations where factors such as culture, types of services/programs offered, demographic of clients considering equity, diversity, and inclusion principles may impact the generalisability of findings. This includes evaluating training effectiveness in low to middle income countries which have different systems of workforce training. Second, current evaluation of training effectiveness did not assess whether practitioners implemented engagement strategies in practice or whether actual rates of father engagement improved following training participation. Future research should examine the performance and impact dimensions of Kirkpatrick’s hierarchy of training evaluation [42] as part of implementation, including observations of practitioners’ behaviour to provide multiple-informant assessment of training effectiveness. Third, estimates of training effects may be biased by self-selection as participation was voluntary and practitioners willing to participate in research and training directed at father engagement may not be representative of the diversity in practitioners within organisations. Self-selection bias could be minimised by implementing online training across an organisation allowing service-level evaluation of changes in father attendance and participation from training.

Finally, future research should extend beyond immediate pre- to post-training evaluations to examine the longer-term effects of training. The previous study in Australia identified minor deterioration in self-reported competency and confidence in engaging fathers from post-training to 3-month follow-up assessment, which was not observed in face-face training [8]. Future research should evaluate longer term outcomes such as 3-month and 1-year follow-up assessments to determine whether deterioration in effectiveness is observed among other practitioners as well as whether online training may require additional support (e.g. booster sessions) to ensure the training effects are maintained over the longer term. This could include evaluating any additional gains of additional support beyond the standard online training program to determine whether incurring costs of support is justified.

Meanwhile, the results of the current study provide preliminary evidence that online training in father engagement competencies can contribute to global workforce development in improving practitioners’ skills in engaging fathers in family-based interventions to optimise treatment outcomes. Training effectiveness associated with the online training program was similar across countries and participants reported high levels of satisfaction with the training program. Qualitative feedback suggests adaption may be required to increase fit of the training program in different local contexts. However, individual difference in contexts may alternatively be addressed via effective implementation in standard training pathways in workforce development. Notwithstanding these considerations, the online ‘Engaging Fathers in Parenting Programs’ training represents a promising direction to overcome significant barriers in workforce development in father engagement competencies in the community.

## Summary

Knowledge and skills regarding the clinical engagement of fathers are core competencies for practitioners delivering family-based interventions, yet access to training in these competencies is often limited. Results from the current study which examined the feasibility of educating practitioners internationally in father engagement using an online training program indicated that participation could improve practitioner confidence and competence in engaging fathers in countries different from the one the original program was developed. Practitioners also reported high levels of training satisfaction. Notable recommendations to enhance relevance in local contexts were to provide local resources and increase representation of diversity in the training. However, further analysis is required to determine how to optimally deliver learning modules that target local cultural variations. Meanwhile, the findings provide preliminary evidence that online training in father engagement competencies can contribute to global workforce development in improving practitioners’ skills in engaging fathers in family-based interventions.

## Supplementary Information

Below is the link to the electronic supplementary material.

Supplementary file1 (DOCX 99 KB)
