# Supplementary material for: Global Workforce Development in Father Engagement Competencies for Family-Based Interventions Using an Online Training Program: A Mixed-Method Feasibility Study
Source: Child Psychiatry Hum Dev. 2021 Nov 20;54(3):758–69. doi: 10.1007/s10578-021-01282-8 (PMC10140122; doi:10.1007/s10578-021-01282-8)
Supplement: Supplementary file 1 — Supplementary file1 (DOCX 99 KB) [file 10578_2021_1282_MOESM1_ESM.docx]

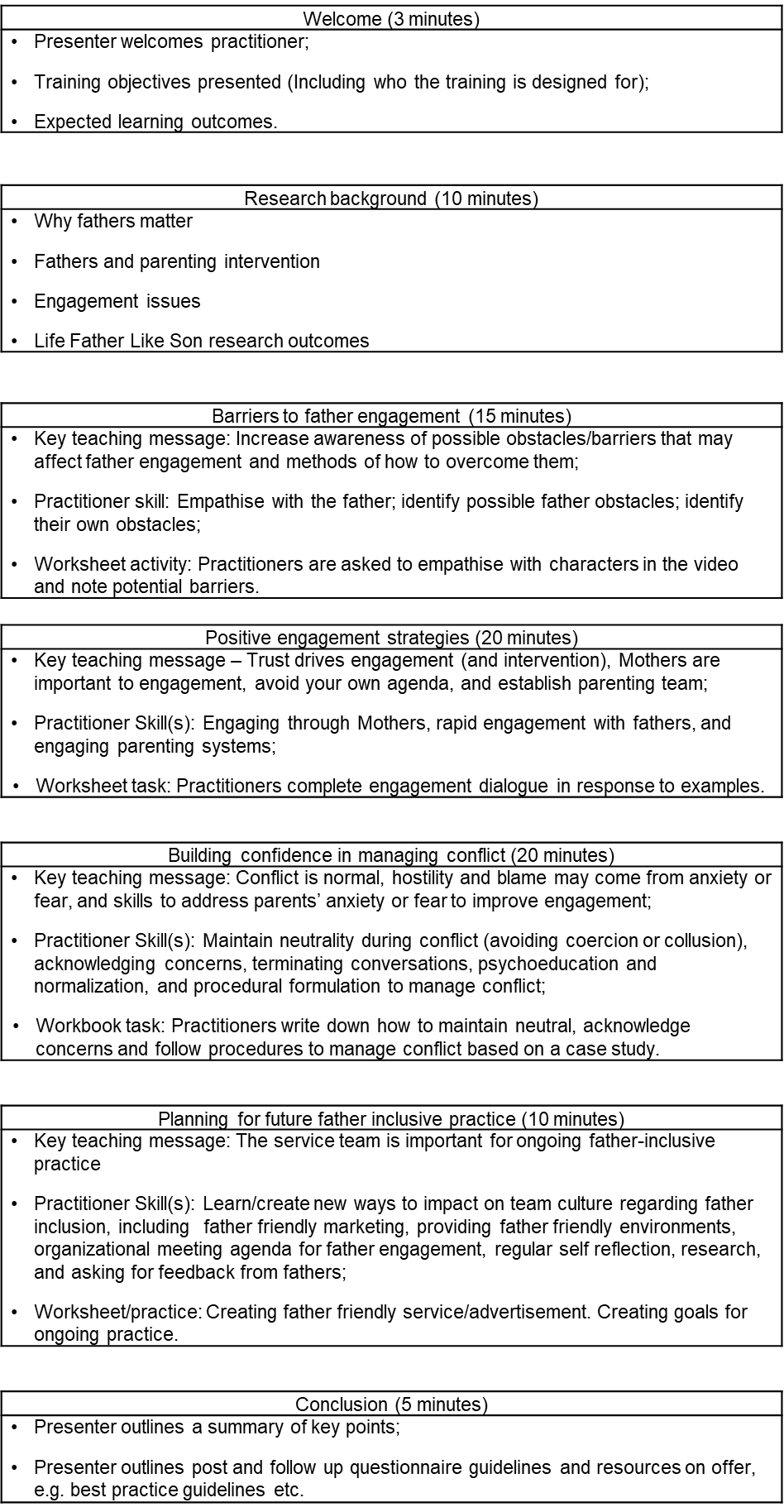


*Figure 1*. Content for online ‘Engaging Fathers in Parenting Programs’ training package.

*Note.* ‘Like Father Like Son’ project refers to research funded by the Movember Foundation.

**Supplementary Table 1**

*Coding of ‘profession’ response set to identify health and care professionals for benchmarking analysis.*

|  | **Health and care professional = yes** | **Health and care professional = no** |
| --- | --- | --- |
| Item 3 What is your profession? | Profession = 1 ‘Psychologist’  Profession = 2 ‘Social worker’  Profession = 3 ‘Counsellor’  Profession = 4 ‘Caseworker’  Profession = 5 ‘Family support worker’  Profession = 6 ‘Nurse (including child health nurse, paediatric nurse etc)  Profession = 7 ‘Psychiatrist’  Profession = 8 ‘Paediatrician’  Profession = 9 ‘General practitioner’  Profession = 10 ‘Occupational therapist’ | Profession = 11 ‘Director/manager’  Profession = 12 ‘Administrative work’ |
| Item 3 What is your profession? Response ‘Other’ | Art therapist  Child advocate worker  Child development specialist  Child and youth worker  Clinical psychology trainee/resident  Community worker  Disability worker  Education mental health practitioner (trainees included)  Midwife  Physiotherapist  Psychotherapist/therapist/CBT therapist  Psychological wellbeing practitioners (trainees included)  Speech pathologist  Youth worker | Church/Clergy/Pastor  Coach/Life coach/Facilitator  Community development worker  Community hub coordinator  Domestic and family violence practitioner  Family dispute resolution  Indigenous coordinator  Librarian  Operations/Program coordinator  Parent/Carer  Parenting consultant  Play specialist  Project/Policy officer or coordinator  Researcher/Research officer  Student (not defined)/Research student (e.g., PhD student)  Support group organiser  Teacher/Educator  Volunteer |

**Supplementary Table 2**

*Thematic analysis of practitioners’ experience of the training program.*

|  |  | Example quotes | | |
| --- | --- | --- | --- | --- |
| Main Theme | Subthemes | Example 1 | Example 2 | Example 3 |
| Delivery method and format (positive) |  | *The format was really good, I liked completing the workbook alongside the training video. The delivery was really good. I liked the video roleplays between the theory sections.* | *I particularly liked the format of being able to watch a scenario, reflect (incorporating new knowledge) and then think about how I would change my future practice accordingly* | *The delivery was really good* |
|  | Combination of resources | *I enjoyed the mix of activities between videos, slides, a person talking and reflecting in the workbook* | *Having a workbook to follow at the time of the video presentation means I can also go back and reflect and remind myself of the key messages in the future* | *Having the workbook and videos included helped keep the training engaging* |
|  | Helpful role play videos | *It was good to have video examples of the ideas in practice* | *The example videos and workbook questions linked to them were very helpful as it showed you what is likely to happen with fathers and how they feel, giving us a good representation of how we should handle those situations, increasing my confidence* | *I particularly liked the format of being able to watch a scenario, reflect (incorporating new knowledge) and then think about how I would change my future practice accordingly* |
| Delivery method and format (negative) | Format created challenges to engage | *Quite a lot to fill in on the form, can be overwhelming* | *It felt like quite a long video, perhaps a few interactive activities would have helped my engagement* | *It would be better delivered in person, it is harder to concentrate over video* |
|  | Preference for face-face training | *I prefer face to face training* | *I found it quite difficult to stay focused, this is due to my preferred learning style - workshops in face to face form would be valuable* | *It’s hard to complete online training without the possibilities of interaction with the trainer* |
| Training content (positive) |  | *All of the training was relevant and helpful* | *All was extremely useful* |  |
|  | Usefulness of engagement strategies | *The establishment of the 'parenting team' was very useful* | *The overview of the research, and the positive engagement strategies were most helpful. I liked the definition of the parenting team* | *How to deal with conflict and how to engage parents who are reluctant* |
|  | Implications for practice | *Positive engagement strategies will be very useful to use in my work with families* | *It was helpful in the sense I will now work harder to engage two parents to ensure positive outcomes for the child.* | *This will impact my daily work with families and has built my confidence in handling difficult situations with families* |
| Training content (negative) | Content development | *The conflict represented in the training was quite mild; it would have been useful to learn how to manage more severe conflict where it had escalated* | *Explore more barriers that fathers face* |  |
| Future training development |  | *I'm not sure that it needs adaptation* | *I think it can be easily adapted for the UK with very little changes* | *I don't think it needs to be adapted. I found it easy to adapt to and the context was very relatable* |
|  | Methods for adapting training | *Firstly, the program is very adaptable the content very much could fit within our context with obvious changes around incorporating locally-based articles* | *Links to local resources/websites* | *Using local data and examples of existing practice* |
|  | Diversity | *Be reflective of the hugely diverse range of fathers we work with as the videos could be hugely offensive. Strategies are fine but for all parents not gender defined* | *Be culturally sensitive, not just towards race. There are fathers who suffer their own mental health difficulties, fathers who engage in crime and or drug use, fathers who aren’t working, fathers who are disabled* | *Maybe include differences that we experience, such as cultural factors, religious view. Identified different parental teams, but did not provide examples of how this may work e.g. two dads, two moms* |
